# Spoofing Attack Results Determination in Code Domain Using a Spoofing Process Equation

**DOI:** 10.3390/s19020293

**Published:** 2019-01-12

**Authors:** Beomju Shin, Minhuck Park, Sanghoon Jeon, Hyoungmin So, Gapjin Kim, Changdon Kee

**Affiliations:** 1 Institute of Advanced Aerospace Technology, School of Mechanical and Aerospace Engineering, Seoul National University, 1 Gwanak-ro, Gwanak-gu, Seoul 08826, Korea; bjshin1984@snu.ac.kr (B.S.); kaito1125@snu.ac.kr (M.P.); 2 Datalab, Kakao Mobillity Corp., 13F, Alphadom Tower, 152, Pangyoyeok-ro, Bundang-gu, Seongnam-si, Gyeonggi-do 13529, Korea; hoon.jeon@kakaomobility.com; 3 Agency for Defense Development, Daejeon 34186, Korea; hmso@add.re.kr (H.S.); ilovebach@add.re.kr (G.K.)

**Keywords:** GNSS spoofing, GNSS receiver, delay lock loop

## Abstract

When a user receiver is tracking an authentic signal, a spoofing signal can be transmitted to the user antenna. The question is under what conditions does the tracking point of the receiver move from the authentic signal to the spoofing signal? In this study, we develop a spoofing process equation (SPE) that can be used to calculate the tracking point of the delay lock loop (DLL) at regular chip intervals for the entire spoofing process. The condition for a successful spoofing signal is analyzed using the SPE. To derive the SPE, parameters, such as the signal strength, sweep velocity, loop filter order, and DLL bandwidth are considered. The success or failure of a spoofing attack is determined for a specific spoofing signal using the SPE. In addition, a correlation between each parameter for a successful spoofing attack could be obtained through the SPE. The simulation results show that the SPE performance is largely consistent with that of general DLL methods, even though the computational load of SPE is very low.

## 1. Introduction

The security and safety aspects of global navigation satellite systems (GNSSs) have been receiving significant attention from researchers and the general public, because the use of GNSSs has been increasing in modern society [1]. Because the power of a GNSS signal coming from the ground is very low, the signal is exposed to different types of radio interferences [2]. Moreover, in contrast to military signals, safety and security issues are not considered for civilian GNSS signals. A civilian signal is not encrypted, and the details of such a signal are open [3]. In other words, anyone can intentionally transmit a fake signal to deceive the user.

Some of the types of intentional interferences include jamming, meaconing, and spoofing [4,5,6]. The aim of jamming is to prevent a user from receiving the authentic signal by transmitting another signal with a significantly greater power than that of the authentic signal. A meaconing attack involves transmitting another signal collected at a different location or time. If a meaconing attack is successful, the receiver would end up providing navigation information, such as the location and time, at which the meaconing signal was collected. The most dangerous type of interference is a spoofing attack. If the receiver captures a spoofing signal, the navigation solution can be controlled by the spoofer [7].

There are two main technological approaches in spoofing studies: spoofing attacks and anti-spoofing techniques. Many spoofing attack tests have been conducted over the past few years. A portable GPS spoofer was developed, and a spoofing attack test was demonstrated for a target receiver [8]. Although this experiment was conducted with a very short distance between the spoofer and the target receiver, it was possible to develop a practical spoofer with low cost. Moreover, successful spoofing tests were carried out against an unmanned aerial vehicle [9], a ship [10], and a mobile device [11]. These studies have shown that spoofing attacks could be executed in real situations. Moreover, many anti-spoofing techniques have been studied for receiver security and safety. A maximum likelihood estimation-based positioning technique was applied to the detection of spoofing signals and correction of navigation solution [12]. In another study, a cross-correlation approach between two GNSS receivers was used to detect the spoofing signal [13]. In Ref [14], an extended coupled amplitude delay lock loop (DLL) architecture was applied to spoofing detection. A pseudorange difference-based anti-spoofing algorithm was introduced [15]. In Ref [16], spoofing detection was performed using a machine learning algorithm such as a neural network. In other studies, antenna-aided techniques [17] and inertial measurements unit-aided techniques [18] have been developed.

Although the aforementioned studies report on spoofing attacks and anti-spoofing techniques, few have analyzed the conditions and circumstances required for a successful spoofing attack. In [19], the spoofing attack results were presented considering the time, position, and power offset. However, only the effects of the spoofing parameters on the spoofing attack results were studied. In [9], a spoofing signal with a 10 dB greater power than that of the authentic signal was transmitted to successfully deceive a drone. However, to avoid as much as possible the detection of a spoofing signal at the victim receiver, it is better to transmit the signal with the minimum power possible for a successful spoofing attack.

In this paper, we analyzed the conditions for a successful spoofing attack in the code domain. The spoofing parameters considered in this study are the spoofing signal strength, spoofing sweep velocity (Doppler offset), DLL order, and bandwidth. With the increase in the spoofing signal strength or DLL bandwidth, the probability of a successful spoofing attack increases. If the sweep velocity increases, the probability of a successful spoofing attack is reduced because of the increase in the Doppler offset between the authentic signal and the spoofing signal. However, for a specific spoofing signal, it is difficult to determine whether a spoofing attack would be successful when the bandwidth is more than a certain level. It is also difficult to determine the correlation between each parameter for a successful spoofing attack. In this research, we develop a spoofing process equation (SPE) for the entire spoofing process. Generally, to determine whether a specific spoofing signal would be successful, it is necessary to perform an iterative DLL calculation during the entire spoofing process. The concept of the SPE is to reduce the number of iterative DLL tracking calculations during the spoofing process by increasing the integration time. Moreover, we express the entire DLL calculation process in the form of an nth order polynomial. The spoofing attack results could be obtained in one single calculation through the SPE. The following are the contributions of this study:

- We develop an SPE that can be used to express the entire spoofing process in the form of an nth order polynomial.
- We obtain the spoofing results in one single calculation using the SPE and determine the correlation between each parameter based on the boundary line which distinguishes between successful and unsuccessful spoofing attacks.
- For a particular receiver, the minimum power of a spoofing signal for a successful spoofing attack could be estimated via the SPE.

The remainder of this paper is organized as follows: Section 2 introduces the auto correlation function (ACF) models of the authentic and spoofing signals. Section 3 presents simulations of a spoofing scenario using the ACF models. Section 4 presents the derivation process of the SPE. Section 5 shows the simulation results obtained using the SPE. Finally, the discussions and conclusions are provided in Section 6 and Section 7, respectively.

## 2. Authentic and Spoofing Signal ACF Model

**Figure 1:**
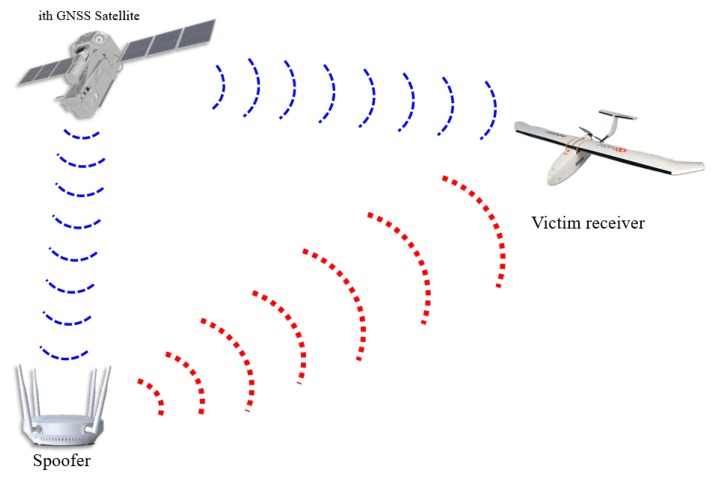
Illustration of a spoofing scenario.

In this section, the authentic and spoofing signal models are presented. To generate a spoofing signal aligned with the authentic signal, the spoofer should estimate the position and velocity of the target receiver. Figure 1 shows a brief illustration of a spoofing scenario. First, the spoofer estimates the position and velocity of the victim receiver using radar [6]. The spoofer can then calculate the aligned spoofing signal by compensating for the spoofer processing delay and transmission delay. Moreover, the power of the spoofing signal should be greater than that of the authentic signal. Therefore, it is necessary to compensate for the propagation loss depending on the distance between the spoofer and the victim receiver. The signal received at the victim receiver antenna can be represented using a complex baseband model as follows:

(1) $$s(t)=C[t-\tau_{a}(t)]\exp(j\phi_{a}(t))+\sqrt{P_{s}}C[t-\tau_{s}(t)]\exp(j\phi_{s}(t))+n(t).$$

where,

- s(t) denotes the total received signal;
- C denotes the pseudorandom code;
- $\tau_{a}(t)$ is the code phase of the authentic signal;
- $\tau_{s}(t)$ is the code phase of the spoofing signal;
- $P_{s}$ is the spoofing power advantage;
- $\phi_{a}(t)$ is the carrier phase of the authentic signal;
- $\phi_{s}(t)$ is the carrier phase of the spoofing signal;
- n(t) is the complex zero-mean white Gaussian noise (AWGN).

**Figure 2:**
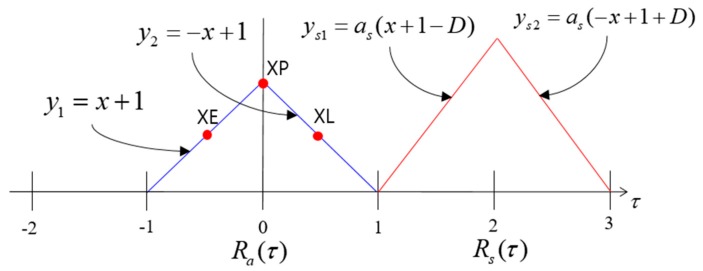
ACFs of the authentic and spoofing signal models.

In the receiver, a correlation process is implemented to track the input signal s(t). Figure 2 shows the ACF model of s(t). The blue triangle indicates the ACF of the authentic signal, whereas the red triangle indicates the ACF of the spoofing signal. The horizontal axis represents the chip offset, and the vertical axis represents the normalized correlator output (the amplitude of the authentic signal is 1). The parameters, shown in Figure 2, can be expressed as follows:

(2) $$\begin{array}{l} y_{1}=\{\begin{array}{c} x+1,\;-1<\tau \leq 0\\ 0,\;\mathrm{else} \end{array}\\ y_{2}=\{\begin{array}{c} -x+1,\;0<\tau <1\\ 0,\;\mathrm{else} \end{array}\\ R_{a}(\tau )=y_{1}+y_{2}\\ D=\tau_{s}-\tau_{a}\\ y_{s1}=\{\begin{array}{c} a_{s}(x+1-D),\;-1+D<\tau \leq D\\ \;0,\;\mathrm{else} \end{array}\\ y_{s2}=\{\begin{array}{c} a_{s}(-x+1+D),\;D<\tau <1+D\\ \;0,\;\mathrm{else} \end{array}\\ R_{s}(\tau )=y_{s1}+y_{s2}\\ R(\tau )=R_{a}(\tau )+R_{s}(\tau ) \end{array}$$

where:

- $y_{1}$ indicates the left line of the ACF of the authentic signal;
- $y_{2}$ indicates the right line of the ACF of the authentic signal;
- $R_{a}(\tau )$ is the ACF of the authentic signal;
- D is the difference in the code phases between spoofing and authentic signals;
- $y_{s1}$ indicates the left line of the ACF of the spoofing signal;
- $y_{s2}$ indicates the right line of the ACF of the spoofing signal;
- $a_{s}$ is the slope of the ACF of the spoofing signal;
- $R_{s}(\tau )$ is the ACF of the authentic signal;
- R(τ) is the ACF of the total signal;
- *XE* is the accumulation result with the replica code separated 0.5 chip early;
- *XP* is the accumulation result with the replica code;
- *XL* is the accumulation result with the replica code separated 0.5 chip late.

The *XP* could be written as [20]:

(3) $$\begin{array}{l} XP[n]=R(\Delta \tau_{a}[n])\cdot \frac{\sin(\pi \Delta f_{a}[n]\cdot T)}{\pi \Delta f_{a}[n]\cdot T}\exp(j\Delta \phi_{a}[n])\\ \;+R[\Delta \tau_{s}[n]]\cdot \frac{\sin(\pi \Delta f_{s}[n]\cdot T)}{\pi \Delta f_{s}[n]\cdot T}\exp(j\Delta \phi_{s}[n]) \end{array}$$

where:

- $\Delta \tau_{a}[n]$ is the code phase difference between the local replica and the authentic signal;
- $\Delta \tau_{s}[n]$ is the code phase difference between the local replica and the spoofing signal;
- $\Delta f_{a}[n]$ is the Doppler frequency difference between the local replica and the authentic signal;
- $\Delta f_{s}[n]$ is the Doppler frequency difference between the local replica and the spoofing signal;
- $\Delta \phi_{a}[n]$ is the carrier phase difference between the local replica and the authentic signal;
- $\Delta \phi_{s}[n]$ is the carrier phase difference between the local replica and the spoofing signal.

In the next section, we explain the change in the replica code phase, τ, depending on the success or failure of a spoofing attack. In our simulation, we assume that the code phase and Doppler frequency of the replica are perfectly aligned with the authentic signal before the spoofing signal approaches. This implies that $\Delta \tau_{a}[1]$ and $\Delta f_{a}[1]$ are zero.

## 3. Spoofing Scenario Simulation Using ACF Model

**Figure 3:**
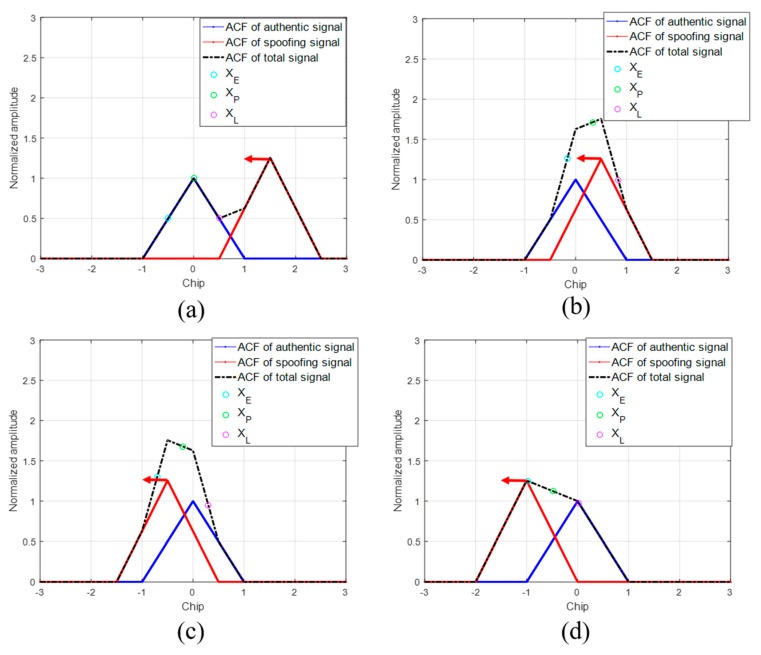
ACF variation with respect to the difference in the code phases between the authentic and spoofing signals. (**a**) D = 1.5; (**b**) D = 0.5; (**c**) D = −0.5; (**d**) D = −1.

Using the ACF models explained in Section 2, we conduct the spoofing simulation. We assume that the authentic signal is stationary and that the spoofing signal is moving from right to left with a static velocity. This simulation is done without noise. In general, the DLL discriminator is used to calculate the feedback output using *XE* and *XL* and thereby track the incoming signal. The replica code phase gradually aligns with the code start point of the incoming signal during DLL code tracking. In our spoofing simulation, the DLL initially tracks the authentic signal. When the spoofing signal approaches and overlays with the authentic signal, the ACF changes. Figure 3 shows the sequential ACF variation during the spoofing simulation. The green circle indicates the *XP*, which is the prompt of the DLL. The position of *XP* in each figure is different. The position of *XP* is determined by the shape of the ACF and the positions of previous *XP* and DLL settings.

**Figure 4:**
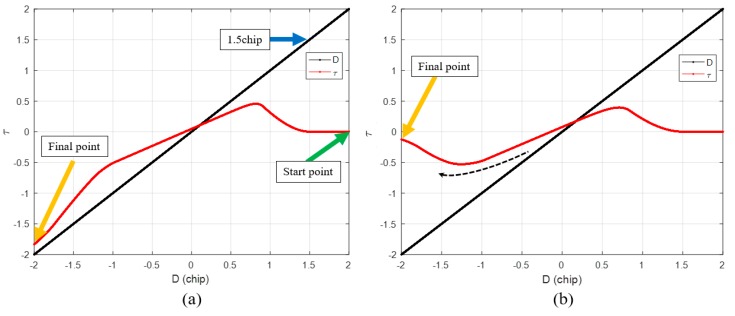
Calculated histories of the local replica code phase in case of (**a**) successful spoofing attack; (**b**) spoofing attack failure.

Figure 4a,b show the τ histories of the two spoofing simulations. The only difference between the two simulations is the DLL bandwidth. In Figure 4, the black lines indicate the code phase distance between the authentic and spoofing signals. Because the authentic signal is fixed at zero, this can be considered the position of the spoofing signal relative to the authentic signal in the code domain. We now focus on Figure 4a. The green arrow indicates the start point of τ. At the start of the simulation, τ is zero. The spoofing signal approaches the authentic signal from a distance of two chips. When the spoofing signal reaches a distance of 1.5 chips, τ starts to gradually increase, as the spoofing signal starts to affect *XL*. A blue arrow indicates the point where τ is increasing. The peak point of the total ACF R(τ) is always the same as that of the spoofing ACF $R_{a}(\tau )$. Therefore, τ moves to the peak point of R(τ) until the discriminator output becomes zero. After the spoofing signal passes the authentic signal, the peak point of R(τ) is located on the negative side, as shown in Figure 3c. Finally, τ follows the spoofing signal. The orange arrow represents the final value of the τ. In the case of Figure 4a, τ follows the spoofing signal, and therefore, the spoofing attack is successful. Figure 4b shows the other spoofing simulation case. In Figure 4b, the final value of τ returns to zero. τ seems to chase the spoofing signal, as indicated using the dotted black line, but eventually returns to its location. This implies that the spoofing attack is a failure. The difference between the two simulations is that the DLL bandwidth. The DLL bandwidth is 5 Hz in the first simulation, whereas it is 3 Hz in the second simulation. As shown in the simulation results, the greater the bandwidth of the receiver, the more vulnerable it is to a spoofing attack. Moreover, the higher the strength of the spoofing signal, the higher is the probability of a successful spoofing attack. The faster the spoofing signal sweeps, the more likely it is that the spoofing attack will fail. Table 1 lists the changes in the spoofing attack results with respect to increases in the bandwidth, signal strength and sweep velocity. However, it is difficult to determine how strong a signal should be for a successful spoofing attack. It is also difficult to obtain a correlation between the different parameters for a successful spoofing attack.

## 4. Development of Spoofing Process Equation

### 4.1. Conventional Approach for τ Calculation

*XP* is calculated through DLL using the ACF and previous *XP*. The first-order DLL can be expressed as follows:

(4) $$\begin{array}{l} \Delta \tau [n]=\frac{XE[n]-XL[n]}{2}\\ \tau [n+1]=\tau [n]-\omega_{0}\cdot T\cdot \Delta \tau [n]\\ \omega_{0}=\frac{B}{4} \end{array}$$

where Δτ, T, and B indicate the discriminator output, integration time, and DLL bandwidth, respectively. In general, the spoofing attack results can be obtained by determining which signal the DLL is tracking after the spoofing signal completely sweeps the authentic signal. In other words, if the integration time of the receiver is 1 ms, it is necessary to repeatedly calculate the equation thousands of times to obtain the spoofing attack results. This calculation can be expressed as follows:

(5) $$\begin{array}{l} k=\frac{\omega_{0}\cdot T}{2}\\ \tau [2]=\tau [1]-\omega_{0}\cdot T\cdot \Delta \tau [1]=\tau [1]-k\cdot \{R_{1}(\tau [1]-\frac{1}{2})-R_{1}(\tau [1]+\frac{1}{2})\}\\ \tau [3]\;=\tau [2]-\omega_{0}\cdot T\cdot \Delta \tau [2]=\;\tau [2]-k\cdot \{R_{2}(\tau [2]-\frac{1}{2})-R_{2}(\tau [2]+\frac{1}{2})\}\\ \;.\\ \;.\\ \;.\\ \tau [n]=\tau [n-1]+\omega_{0}\cdot T\cdot \Delta \tau [n-1]=\;\tau [n-1]-k\cdot \{R_{n-1}(\tau [n-1]-\frac{1}{2})-R_{n-1}(\tau [n-1]+\frac{1}{2})\}\\ \tau [n]=\tau [1]-(n-1)\cdot k\cdot \sum_{m=1}^{n-1}\left\{R_{m}(\tau [m]-\frac{1}{2})-R_{m}(\tau [m]+\frac{1}{2}\right)\} \end{array}$$

For a specific spoofing attack scenario, a lot of computations are required to calculate the final replica code phase τ[n]. Moreover, it is necessary to know τ and ACF at all previous epochs. Thus, the final τ value can be written as follows:

(6) $$\tau [n]=f_{\tau }(\tau [1],\;\tau [2],\;\tau [3]\;,\;,,\;\tau {\left[n-1\right]}_{,}R_{1},R_{2},R_{3},,,R_{n-1}).$$

### 4.2. Proposed Approach for τ Calculation

**Figure 5:**
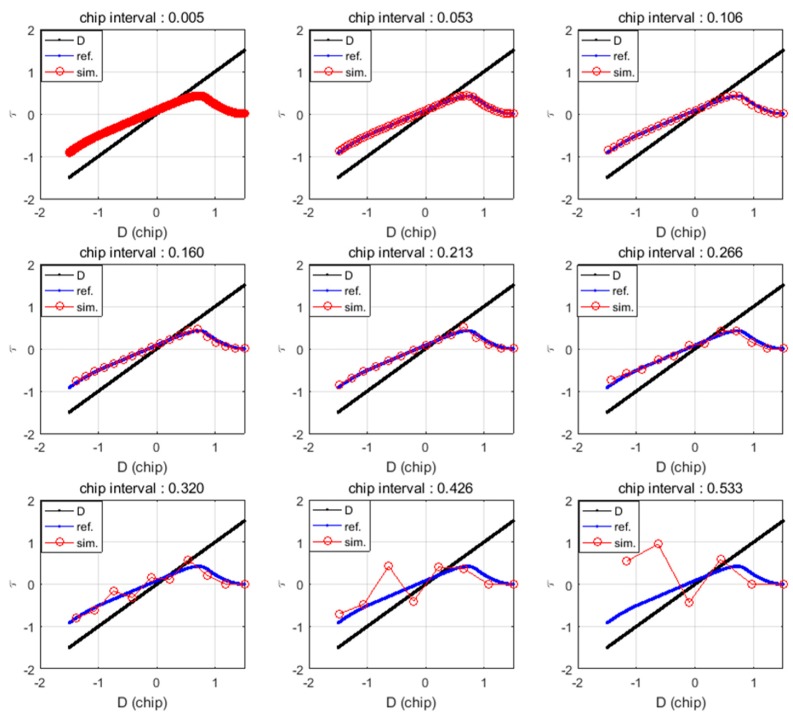
Replica code phase histories for various CI settings. The blue lines indicate the calculated replica code phase values of the original calculation. The red lines indicate the calculated replica code phase with respect to the CI.

In this subsection, we propose a method to compute the spoofing attack results by calculating each epoch at a certain chip interval (CI). The entire spoofing process is summarized in a mathematical equation, i.e., the SPE, and the spoofing results are obtained by one calculation using the SPE. Figure 5 shows the results of the τ estimation with respect to the CI. The blue lines indicate the calculation results of τ per *1* ms. Red circles indicate the calculation results of τ per specific CI. The equation for calculating the time interval (TI) in terms of the chip interval can be expressed as follows:

(7) $$TI=\frac{293}{V_{s}}\cdot CI,$$

where $V_{s}$ denotes the spoofing sweep velocity (m/s), and the number 293 indicates the wavelength of the C/A code in meter-scale. Table 2 lists the calculated TI with respect to each CI in case of the spoofing sweep velocity is 80 m/s. τ error decreases with the decrease in the CI. However, additional calculations are required to estimate the final τ when CI is low. The concept of CI is similar with sampling interval. CI determines how often the SPE calculates the replica code phase. In our research, we set the CI to 0.125 considering the complexity of the equation and τ error.

### 4.3. Spoofing Attack Success or Failure Criteria

**Figure 6:**
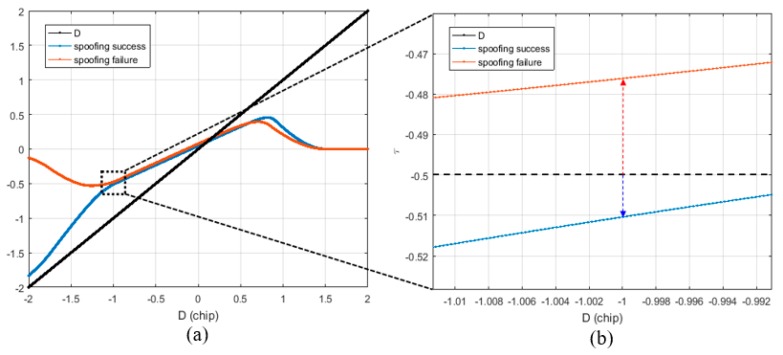
Different τ values at D is −1 according to the spoofing attack success or failure. (**a**) Overall τ histories; (**b**) τ values at D is −1.

Figure 6a shows the τ results of DLL when the spoofing signal sweeps the authentic signal with constant velocity. The blue line indicates the case of a successful spoofing attack, whereas the red line indicates the case of a failed spoofing attack. Generally, the success or failure of a spoofing attack can be determined from the type of signal the DLL tracks when the spoofing signal completely sweeps the authentic signal. In both the simulations, the only difference is the bandwidth of the DLL.

The success or failure of a spoofing attack can be determined by looking at the absolute value of τ at the point where *D* is −1. Figure 6b shows the region enclosed in the black box shown in Figure 6a. If the spoofing attack is successful, the absolute value of τ exceeds 0.5 at the point where *D* is −1, and if it fails, the absolute value of the prompt is lower than 0.5.

**Figure 7:**
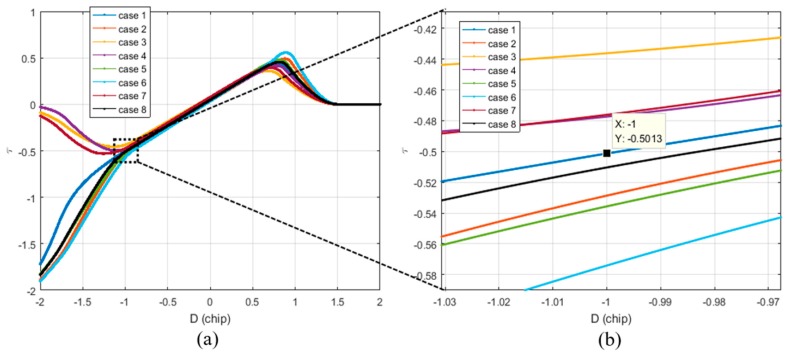
Different τ values at D is −1 according to the various spoofing attack scenarios. (**a**) Overall τ histories; (**b**) τ values at D is −1.

Figure 7a shows the τ estimates for various spoofing parameters listed in Table 3. It is noteworthy that the absolute value of τ at *D* is −1. As shown in Figure 7b, if the absolute value of τ exceeds 0.5 chip when *D* is −1, the DLL tracks the spoofing signal.

**Figure 8:**
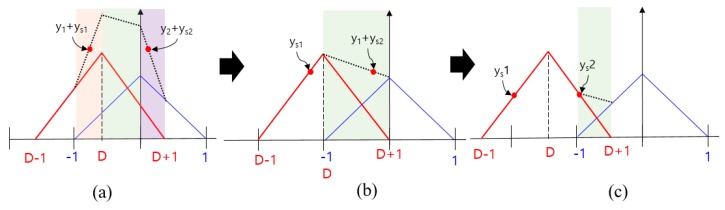
ACF change according to the spoofing signal in case that spoofing signal strength is larger than authentic signal strength. (**a**) −1<D<0; (**b**) D=1; (**c**) D<−1.

The following analysis shows that the criterion used for determining the spoofing result is reasonable. If the spoofing sweep velocity is very low or if the bandwidth is very high in the spoofing simulation, there will be sufficient time or control input for the DLL to track the peak point of the ACF. In this case, the discriminator output would become zero and *XP* would be located at the point where *XE* equals *XL*. Figure 8 shows a series of snapshots where the discriminator output is zero with respect to the ACF. The τ value for the case, shown in Figure 8a, can be derived as follows:

(8) $$\begin{array}{l} XE=y_{1}+y_{s1}\\ =x+1+a_{s}(x+1-D)\\ =(a_{s}+1)x+a_{s}-a_{s}D+1\\ =(a_{s}+1)(\tau -\frac{1}{2})+a_{s}-a_{s}D+1\\ XL=y_{2}+y_{s2}\\ =-x+1+a_{s}(-x+1+D)\\ =-(a_{s}+1)x+a_{s}+a_{s}D+1\\ =-(a_{s}+1)(\tau +\frac{1}{2})+a_{s}+a_{s}D+1\\ XE=XL\\ (a_{s}+1)(\tau -\frac{1}{2})+a_{s}-a_{s}D+1=-(a_{s}+1)(\tau +\frac{1}{2})+a_{s}+a_{s}D+1\\ (a_{s}+1)\tau -\frac{1}{2}(a_{s}+1)+a_{s}-a_{s}D+1=-(a_{s}+1)\tau -\frac{1}{2}(a_{s}+1)+a_{s}+a_{s}D+1\\ 2(a_{s}+1)\tau =2a_{s}D\\ \tau =\frac{a_{s}D}{a_{s}+1} \end{array}$$

Moreover, the τ values for the cases, shown in Figure 8b,c, can be derived in a similar manner as follows:

(9) $$\begin{array}{l} y_{s1}=y_{1}+y_{s2}\\ a_{s}(\tau -\frac{1}{2})+a_{s}-a_{s}d=(-a_{s}+1)(\tau +\frac{1}{2})+a_{s}+a_{s}d+1\\ \tau =\frac{2a_{s}d+\frac{3}{2}}{2a_{s}-1} \end{array}$$

(10) $$\begin{array}{l} y_{s1}=y_{s2}\\ a_{s}(\tau -\frac{1}{2})+a_{s}-a_{s}D=-a_{s}(\tau +\frac{1}{2})+a_{s}+a_{s}D\\ \tau =D \end{array}$$

**Figure 9:**
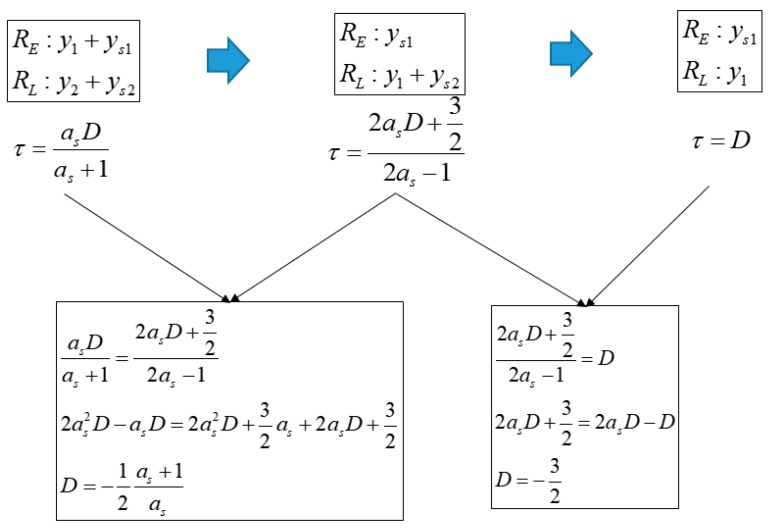
Equation of τ and D in case that the spoofing signal strength is larger than authentic signal and *XE* is same with *XL*.

**Figure 10:**
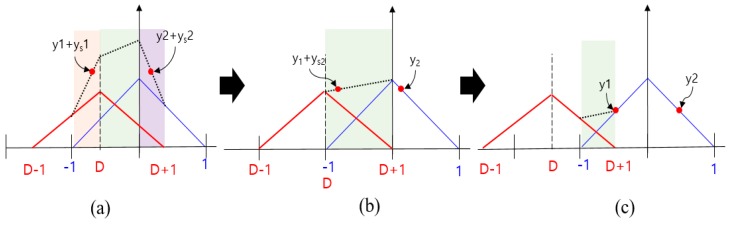
ACF change according to the spoofing signal in case that spoofing signal strength is lower than authentic signal strength. (**a**) −1<D<0; (**b**) D=1; (**c**) D<−1.

Figure 9 shows the summary of Equations (8) to (10). For any ACF, shown in Figure 8, τ can be estimated using $a_{s}$ and *D* when the spoofing sweep velocity is very low or when the bandwidth is considerable. Moreover, it is possible to calculate *D* corresponding to the different equations of τ Figure 10 shows the ACF change with respect to the spoofing signal when the spoofing signal strength is lower than the authentic signal strength. We can derive an equation to calculate τ in the same manner as above. The τ value for the cases, shown in Figure 10a,b,c can be derived as follows:

(11) $$\begin{array}{l} y_{1}+y_{s1}=y_{2}+y_{s2}\\ (a_{s}+1)(\tau -\frac{1}{2})+a_{s}-a_{s}D+1=-(a_{s}+1)(\tau +\frac{1}{2})+a_{s}+a_{s}D+1\\ \tau =\frac{a_{s}D}{a_{s}+1} \end{array}$$

(12) $$\begin{array}{l} y_{1}+y_{s2}=y_{2}\\ (-a_{s}+1)(\tau -\frac{1}{2})+a_{s}+a_{s}D+1=-(\tau +\frac{1}{2})+1\\ \tau =\frac{\frac{3}{2}a_{s}+a_{s}D}{a_{s}-2} \end{array}$$

(13) $$\begin{array}{l} y_{1}=y_{2}\\ (\tau -\frac{1}{2})+1=-(\tau +\frac{1}{2})+1\\ \tau =0 \end{array}$$

**Figure 11:**
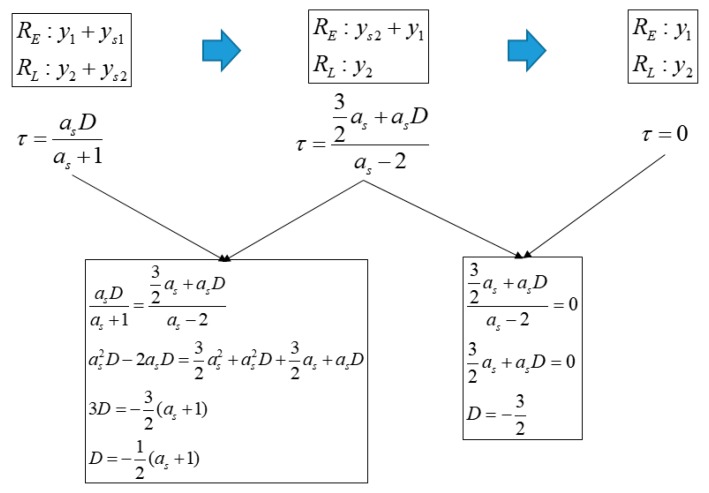
Equation of τ and D in case that the spoofing signal strength is lower than authentic signal and *XE* is same with *XL*.

**Figure 12:**
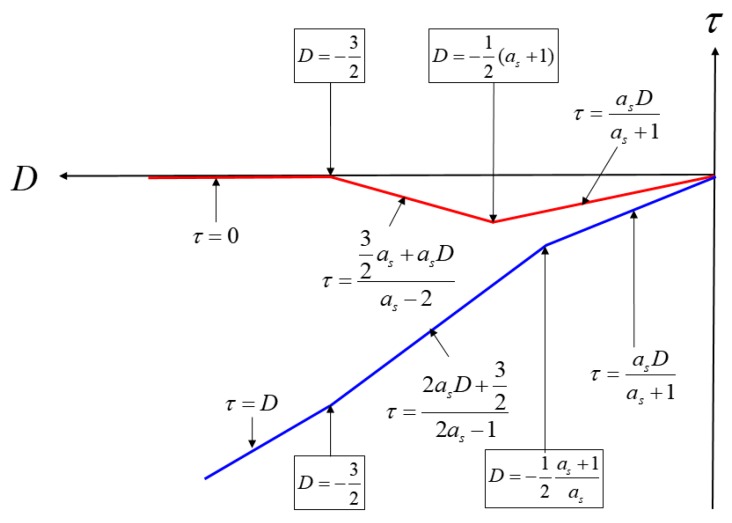
Summary of Equations (8) to (13).

**Figure 13:**
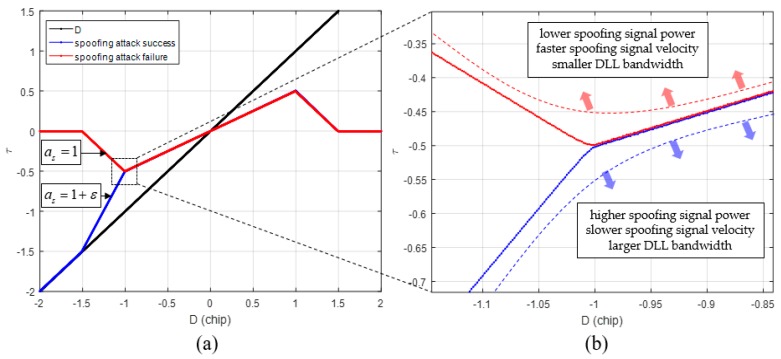
(**a**) τ histories with respect to spoofing signal power in case that the spoofing sweep velocity is infinitely slow; (**b**) τ value change with respect to spoofing parameter change.

Figure 11 shows the summary of Equations (11) to (13). Figure 12 shows a graphical representation of Equations (8) to (13). The blue lines indicate spoofing attack success, whereas the red lines indicate spoofing attack failure. If $a_{s}$ is greater than 1, τ follows the blue line, and if $a_{s}$ is lower than 1, it follows the red line. When $a_{s}$ is 1, *D* at the time of transition, from (b) to (c) in Figure 8, is −1, and the absolute value of τ becomes 0.5. In case that the spoofing sweep velocity is infinitely slow or the DLL bandwidth is infinitely large, the condition for a successful spoofing attack is that the power of the spoofing signal sets in greater than that of the authentic signal. If the spoofing signal power is a little greater than the authentic signal power, the replica code phase is lower than −0.5 at D is −1. If the spoofing signal power is a little smaller than the authentic signal power, the replica code phase is higher than −0.5 at D is −1. Thus, we could regard that the τ value is a boundary value when D is −1. This implies that the absolute value of τ should exceed 0.5 chip before D approaches −1 for a successful spoofing attack. Figure 13a shows the τ histories with respect the spoofing signal power. ε indicates a very small positive value. Slope of each line is differently changed after D exceeds −1. Figure 13b shows the τ value change with respect the spoofing parameters in the real scenario.

### 4.4. Derivation of SPE

Assuming that the spoofing signal shifts from right to left, the spoofing signal affects the DLL discriminator when *D* approaches within 1.5 chip. Moreover, to determine the spoofing results, we only need to calculate τ until *D* reaches −1. Therefore, if CI is 0.125 chip, it is possible to determine the spoofing attack results by a total of 19 calculations. In Equation (4), all the previous *τ* values and ACF are required to calculate τ[n]. The SPE can be used to calculate *τ* at the point where *D* is −1 to determine whether the spoofing attack is a successful one or not by only one calculation. When setting the CI to 0.125, the ACF variation could be known. However, it is not possible to specify the previous *τ* values. This is because *τ* value at a certain D changes according to the spoofing parameters. However, the range of *τ* can be defined for each D value for *τ* to be close to −0.5 chip when D is −1. The spoofing attack results can be determined by checking whether the absolute value of *τ* at D = −1 exceeds 0.5 chip or not. In our case, *τ*[19] is the final *τ* value. For *τ*[19] to be close to −0.5, *τ*[18] must be in a specific range. Moreover, *τ*[17] must be in a specific range for *τ*[18] to be in the defined range. Thus, we can define each range according to the D value of the entire process. Table 4 lists the range of *τ*[*i*] for each *D*[*i*] where *i* denotes each CI index. There are ACF models of *XE* and *XL* for each D. If each *τ*[*i*] is within the defined range for each *D*[*i*], *τ*[19] will be calculated close to −0.5. The details of the SPE derivation are given in appendix A. Finally, SPE has the following form like:

(14) $$\tau [19]=f(a_{s},Vs,B).$$

The inputs to the SPE are the spoofing signal strength, spoofing sweep velocity and DLL bandwidth. When the calculation of SPE is performed, τ[19] is calculated for D = −1 by just one calculation. The success or failure of the spoofing attack is determined by the absolute value of τ.

## 5. Analysis of SPE Simulation Results

### 5.1. SPE Performance Analysis

To verify the performance of the SPE, we compared the estimated SPE results with the original DLL results. Table 5 presents the various spoofing signal parameters and τ results in the cases of using the original DLL and SPE at *D* = *−*1.

**Figure 14:**
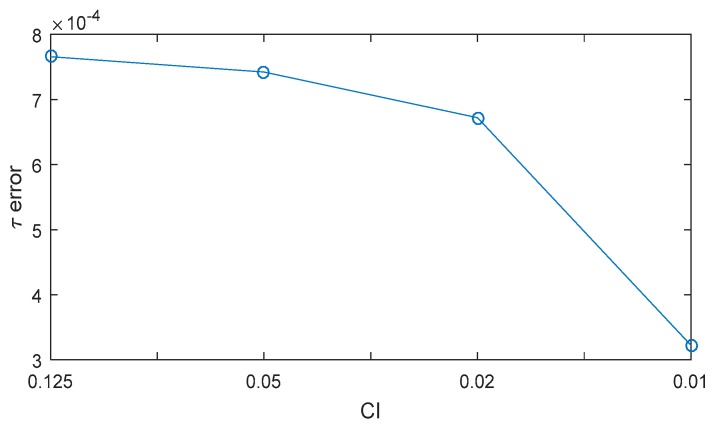
SPE error with respect to CI when D is −1.

$\tau_{1ms}$ indicates the estimated replica code phase obtained using the original DLL, the integration time of which is *1ms*. $\tau_{SPE}$ is the estimated replica code phase obtained using the SPE. The calculation time required by the SPE is significantly lower than that required by the original DLL, because $\tau_{SPE}$ can be calculated in just one calculation using the SPE. We can see that the replica code phase values estimated using the two methods are very similar. Figure 14 shows the $\tau_{SPE}$ errors with respect to CI. The $\tau_{SPE}$ errors decrease with the decrease in CI. Thus, the SPE error is due to the reduction in the number of DLL calculations during the spoofing attack process.

**Figure 15:**
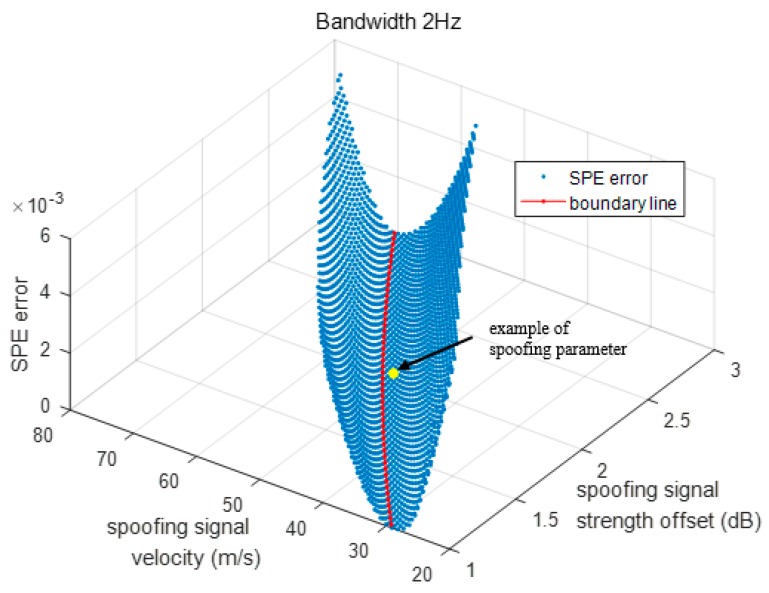
SPE error with respect to the spoofing signal strength and velocity in three dimensions.

Figure 15 shows the error distribution of the SPE with respect to the spoofing signal strength and sweep velocity on a fixed bandwidth. At the yellow point, the values of the spoofing parameters, namely the signal strength offset, sweep velocity, and bandwidth, are 2 dB, 50 m/s, and 2 Hz, respectively. When $\tau_{SPE}$ is calculated for the above set of spoofing parameter values using the SPE, the error in $\tau_{SPE}$ is the Z axis value corresponding to the yellow point. The SPE performance is the best around the boundary line. The success and failure of the spoofing attack can be divided based on the boundary line. 

In other words, $\tau_{SPE}$ of the spoofing parameters on the boundary line is *−*0.5. $\tau_{SPE}$ error increases as the distance from the boundary value and spoofing parameter increases. A large error indicates that the previous τ values are outside the defined range in the $\tau_{SPE}$ calculation process. We define the range of previous replica code phase range at every *D[i]* like Table 4 in order that the final code phase is calculated around the boundary line at *D is* −1. If the previous code phases, τ[1]~τ[18], are deviated from the defined range, SPE would provide inaccurate result.

**Figure 16:**
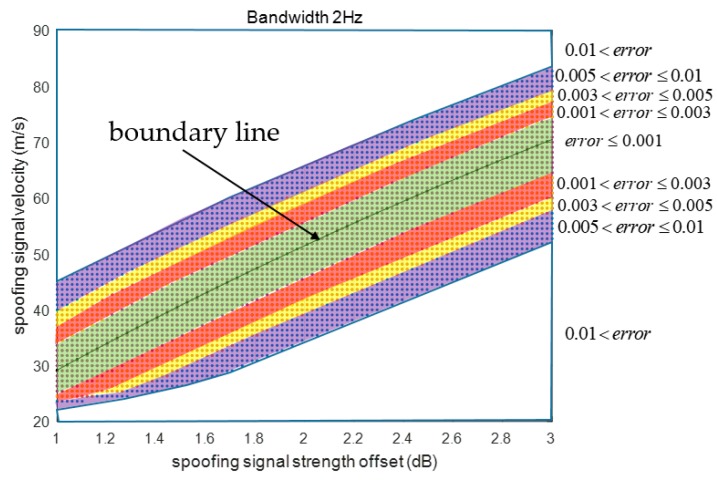
SPE error according to spoofing signal strength and velocity.

Also, it could be explained that the ACF functions used to calculate *XE* and *XL* vary with respect to the already defined *XE* and *XL*. Figure 16 shows the SPE error distribution in two dimensions. The SPE error is lowest around the boundary line. The spoofing parameters are divided using different colors with respect to the SPE error size.

### 5.2. Determination of Boundary Line and Surface Using SPE

The boundary line and surface that divide the spoofing attack success and failure can be estimated using the SPE. The input parameters of the SPE are the spoofing signal strength, spoofing signal sweep velocity, and DLL bandwidth. In Equation (15), if we set each variable as follows:

(15) $$-0.5=f(a_{s},40,2),$$

**Figure 17:**
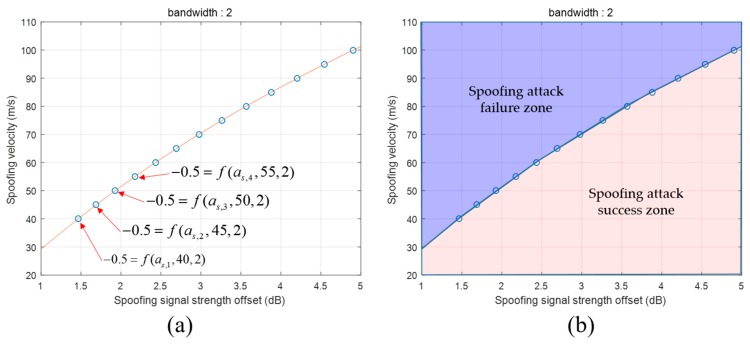
(**a**) $a_{s}$ estimation using SPE; (**b**) Determination of spoofing attack success and failure by boundary line.

Only one variable, i.e., as, remains, and the SPE becomes an equation to calculate as. We use MATLAB solver to obtain ${\tilde{a}}_{s}$ which is an estimated value of $a_{s}$ obtained using Equation (15). This means that the SPE result of the spoofing parameters, (${\tilde{a}}_{s}$, 40, 2), becomes −0.5. Therefore, the spoofing parameter, (${\tilde{a}}_{s}+\varepsilon$, 40, 2), will result in a successful spoofing attack. The other spoofing parameter, (${\tilde{a}}_{s}-\varepsilon$, 40, 2), will result in a failed spoofing attack. ε is a small positive value. Figure 17a shows the boundary line dividing the spoofing attack success and failure zones. We obtain the spoofing signal strength values by fixing the other spoofing parameters and τ. Table 6 presents the estimated ${\tilde{a}}_{s}$ values with respect to the spoofing parameters. The red line in Figure 17a indicates the boundary line. The boundary can be estimated using the spoofing parameters listed in Table 6 as follows:

(16) $$\begin{array}{l} V_{s}=f_{bl}(a_{s})\\ \;=\;p_{1}\cdot a_{s}^{3}+p_{2}\cdot a_{s}^{2}+p_{3}\cdot a_{s}+p_{4} \end{array}$$

where $f_{bl}$ is the function of the boundary line for the DLL bandwidth of 2 Hz. $p_{1}$, $p_{2}$, $p_{3}$, and $p_{4}$ are the coefficients at the boundary line. Moreover, this line expresses the correlation between two parameters for a successful spoofing attack. From Equation (16), we find that as the spoofing signal strength increases, the spoofing attack becomes successful even with a higher sweep velocity.

**Figure 18:**
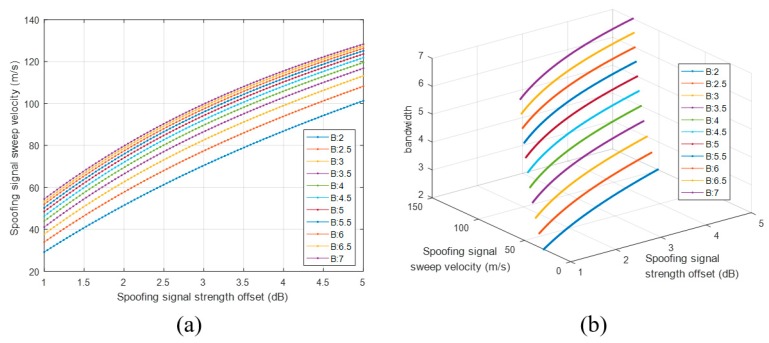
Boundary lines according to the DLL bandwidth (**a**) boundary lines in two dimension; (**b**) boundary lines in three dimension.

The spoofing attack success and failure zone is divided based on the boundary line, as shown in Figure 17b. The zone above the boundary line indicates spoofing attack failure, whereas the zone below indicates spoofing attack success. Figure 18a shows the boundary lines for various DLL bandwidths. We find that the receiver becomes more vulnerable to spoofing attacks as its DLL bandwidth increases. With the increase in the DLL bandwidth, the spoofing attack becomes successful even for a low spoofing signal strength when using a fixed spoofing sweep velocity. Figure 18b shows the boundary lines in three dimensions.

**Figure 19:**
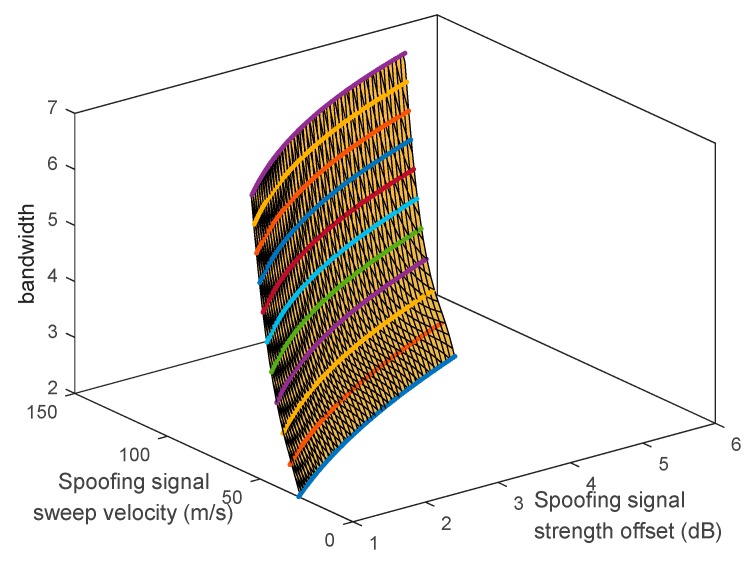
Boundary surface.

The boundary surface can be estimated using the boundary lines, as shown in Figure 19. The boundary surface can be expressed as follows:

(17) $$\begin{array}{l} B=f_{sf}(a_{s},V_{s})\\ \;=c_{1}+c_{2}a_{s}+c_{3}V_{s}+c_{4}a_{s}^{2}+c_{5}a_{s}^{2}V_{s}+c_{6}a_{s}V_{s}^{2}+c_{7}V_{s}^{3}+c_{8}a_{s}^{3}V_{s}+c_{9}a_{s}^{2}V_{s}^{2}+c_{10}a_{s}V_{s}^{3}+c_{11}V_{s}^{4} \end{array}$$

where $f_{sf}$ indicates the function of the boundary surface. $c_{1}\sim c_{11}$ are coefficients of $f_{sf}$. For specific spoofing parameters, the spoofing attack results can be determined using $f_{sf}$. Equation (18) is the case for spoofing attack failure, whereas Equation (19) is the case for spoofing attack success:

(18) $$B>f_{sf}(a_{s},V_{s}),$$

(19) $$B<f_{sf}(a_{s},V_{s}).$$

Table 7 presents the computational complexities of the conventional DLL and SPE in terms of the number of iteration and computational time with respect to the spoofing signal velocity. We can see that the proposed method has much lower computational complexity than that of the conventional DLL. The simulation were performed on a personal computer with Intel Core i7-4790k. In case of the conventional DLL, as the spoofing signal velocity decreases, the computational load increases. In contrast, a consistent computational load is required in case of SPE.

## 6. Discussion

- The SPE can be derived in the same manner regardless of the DLL order. The details of the same are given in Appendix B.
- In this paper, we analyzed the effect of the spoofing signal on the local replica code phase using the SPE. However, for a completely successful spoofing attack, the point of FLL tracking should be moved from the authentic signal to the spoofing signal. In the future, we will focus on spoofing process analysis in the frequency domain.
- Our simulation is conducted without any noise. If noise is added to our simulation, the probability distribution around the boundary line can be obtained using the SPE. The probability of spoofing attack success or failure on the boundary line would be 50%.

## 7. Conclusions

Analyzing the replica code phase variation due to the reception of the spoofing signal is important for developing spoofing attack or anti-spoofing techniques. In this paper, we propose an SPE that can be used to calculate the replica code phase following a spoofing attack and determine whether the spoofing attack is successful using the SPE output. The advantage of the SPE is that it could theoretically create a minimal spoofing signal condition for a successful spoofing attack. The boundary surface dividing the spoofing attack success or failure is obtained using the SPE. The boundary surface shows the correlation of how each spoofing parameter affects the code tracking results. This study is meaningful in that it presents a detailed study about the variation in the replica code phase during a spoofing attack process. We expect that the research results would aid the development of spoofing attack or anti-spoofing techniques.

## Figures and Tables

**Table 1:** Relationship between spoofing parameters and spoofing results.

Parameters	Spoofing Attack Success Probability
Increase in the spoofing signal strength	Increase
Increase in spoofing signal sweep velocity	Decrease
Increase in the DLL bandwidth	Increase

**Table 2:** Time interval calculation according to CI.

Chip Interval (chip)	Time Interval (second)
0.005	0.0183
0.053	0.1941
0.125	0.4578
0.160	0.5860
0.213	0.7801
0.266	0.9742
0.320	1.1720
0.426	1.5602
0.533	1.9521

**Table 3:** Integration time calculation according to CI.

Case	Spoofing Signal Strength Offset (dB)	Sweep Velocity (m/s)	Bandwidth	Spoofing Results	|τ| at *D* = −1
1	1.5	50	3	Success	0.5013
2	1.5	50	5	Success	0.5287
3	1.5	70	3	Failure	0.4362
4	1.5	70	5	Failure	0.4774
5	2	50	3	Success	0.5357
6	2	50	5	Success	0.5741
7	2	70	3	Failure	0.4761
8	2	70	5	Success	0.5104

**Table 4:** Range of τ[i] and ACF model of *XE* and *XL* according to the *D[i]*.

*i*	*D[i]*	Range of τ[i]−0.5	Range of τ[i]+0.5	ACF model of *XE*	ACF model of *XL*
1	1.375	−1~0	0.375~1	y_1_	y_2_ + y_s1_
2	1.25	−1~0	0.25~1	y_1_	y_2_ + y_s1_
3	1.125	−1~0	0.125~1	y_1_	y_2_ + y_s1_
4	1	−1~0	0~1	y_1_	y_2_ + y_s1_
5	0.875	−1~−0.25	−0.125~0.875	y_1_	y_2_ + y_s1_
6	0.75	−0.25~0	0.75~1	y_1_ + y_s1_	y_2_ + y_s2_
7	0.675	−0.375~0	0.625~1	y_1_ + y_s1_	y_2_ + y_s2_
8	0.5	−0.5~0	0.5~1	y_1_ + y_s1_	y_2_ + y_s2_
9	0.375	−0.625~0	0.375~1	y_1_ + y_s1_	y_2_ + y_s2_
10	0.25	−0.75~0	0.25~1	y_1_ + y_s1_	y_2_ + y_s2_
11	0.125	−0.875~0	0.125~1	y_1_ + y_s1_	y_2_ + y_s2_
12	0	−1~0	0~1	y_1_ + y_s1_	y_2_ + y_s2_
13	−0.125	−1~−0.125	0~0.875	y_1_ + y_s1_	y_2_ + y_s2_
14	−0.25	−1~−0.25	0~0.75	y_1_ + y_s1_	y_2_ + y_s2_
15	−0.375	−1~0.375	0~0.625	y_1_ + y_s1_	y_2_ + y_s2_
16	−0.5	−1~−0.5	0~0.5	y_1_ + y_s1_	y_2_ + y_s2_
17	−0.625	−1~0.625	0~0.375	y_1_ + y_s1_	y_2_ + y_s2_
18	−0.75	−1~−0.75	0~0.25	y_1_ + y_s1_	y_2_ + y_s2_
19	−0.875	−1~0.875	0~0.125	y_1_ + y_s1_	y_2_ + y_s2_

**Table 5:** Various spoofing parameters and τ results in case of using original DLL and SPE.

Case	Spoofing Signal Strength Offset (dB)	Sweep Velocity (m/s)	Bandwidth	Reference	Proposed	Error
				$\tau_{1ms}$	$\tau_{SPE}$	$\sqrt{{\left(\tau_{1ms}-\tau_{SEP}\right)}^{2}}$
1	1.5	55	3	−0.4895	−0.4903	0.0008
2	1.5	60	3	−0.4742	−0.4777	0.0035
3	1.5	60	5	−0.5043	−0.503	0.0013
4	1.5	65	5	−0.4927	−0.4931	0.0004
5	2	60	3	−0.5072	−0.507	0.0002
6	2	65	3	−0.4934	−0.4937	0.0003
7	2	65	5	−0.5257	−0.5217	0.004
8	2	70	3	−0.477	−0.4797	0.0027
9	2	70	5	−0.5112	−0.5104	0.0008
10	2.5	65	3	−0.5253	−0.5231	0.0022
11	2.5	80	3	−0.4759	−0.4787	0.0028
12	2.5	80	5	−0.5147	−0.5136	0.0011

**Table 6:** Estimated ${\tilde{a}}_{s}$ values according to the spoofing parameters.

Number	Sweep Velocity (m/s)	Bandwidth (Hz)	${\tilde{a}}_{s}$ (Hz)
1	40	2	1.46
2	45	2	1.69
3	50	2	1.92
4	55	2	2.17
5	60	2	2.42
6	65	2	2.69
7	70	2	2.97
8	75	2	3.26
9	80	2	3.56
10	85	2	3.87
11	90	2	4.20
12	95	2	4.54
13	100	2	4.09

**Table 7:** Computational complexities of conventional DLL and SPE.

Sweep Velocity (m/s)	Conventional DLL		SPE	
	The Number of Iteration	Computational Time (s)	The Number of Iteration	Computational Time (s)
40	18312	10.34	19	0.24
50	14650	8.26	19	0.24
60	12208	6.86	19	0.24
70	10464	5.89	19	0.24
80	9156	5.17	19	0.24

**Figure A1:**
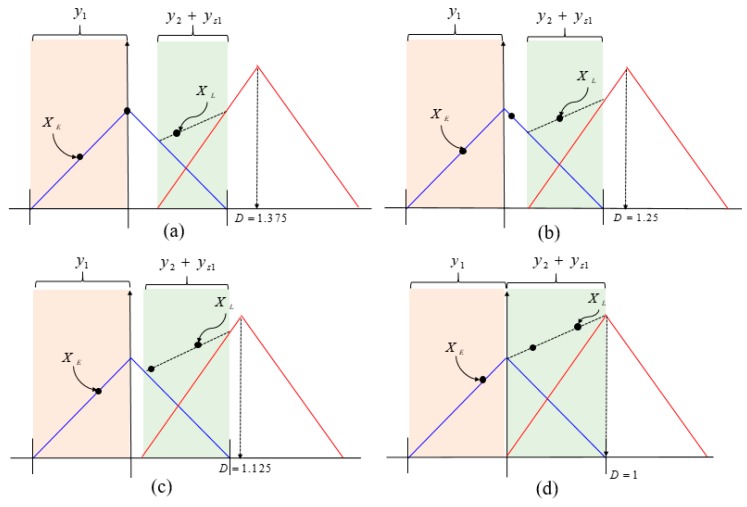
ACF snapshots with *i* from 1 to 4. (**a**) D = 1.375; (**b**) D = 1.25; (**c**) D = 1.125; (**d**) D = 1.

## Appendix A

The details of SPE derivation are as follows. Figure A1 shows the ACF snapshots for *i* ranging from 1 to 4. If τ[i]−0.5 and τ[i]+0.5 are in the defined range, *XE* and *XL* can be calculated using $y_{1}(\tau [i]-0.5)$ and $y_{2}(\tau [i]+0.5)+y_{s2}(\tau [i]+0.5)$, respectively. Thus, first-order DLL could be expressed until i≤5 like:

(A1) $$\tau [i]=\tau [i-1]+k\{(a-2)\tau [i-1]+\frac{3}{2}a_{s}-ad[i-1]\}.$$

Also, τ[1], τ[2], τ[3], and τ[4] can be expressed as follows:

(A2) $$\begin{array}{l} \tau [1]=-k\cdot \{R_{1}(\tau [1]-\frac{1}{2})-R_{1}(\tau [1]+\frac{1}{2})\}\\ \;=-k\cdot \{y_{1}(-\frac{1}{2})-y_{2}(\frac{1}{2})-y_{s1}(\frac{1}{2})\}\\ \;=k(\frac{3}{2}a_{s}-D[1]) \end{array},$$

(A3) $$\begin{array}{l} \tau [2]=\tau [1]-k\{y_{1}(\tau [1]-\frac{1}{2})-y_{2}(\tau [1]+\frac{1}{2})-y_{s1}(\tau [1]+\frac{1}{2})\}\\ \;=k\{(a_{s}-2)(\frac{3}{2}a_{s}-a_{s}D[1])k+(2\frac{3}{2}a_{s}-a_{s}(D[1]+D[2])\} \end{array},$$

(A4) $$\begin{array}{l} \hat{\tau }[3]=k\{{\left(a_{s}-2\right)}^{2}(\frac{3}{2}a_{s}-a_{s}D[1])k+(a_{s}-2)(\frac{3}{2}a_{s}-a_{s}D[1])k\\ +(a_{s}-2)(\frac{3}{2}a_{s}+\frac{3}{2}a_{s}-a_{s}D[2]-aD[2])k+(3\cdot \frac{3}{2}a_{s}-a_{s}(D[1]+D[2]+D[3])\} \end{array},$$

(A5) $$\begin{array}{l} \hat{\tau }[4]=k\{{\left(a_{s}-2\right)}^{3}(\frac{3}{2}a_{s}-a_{s}D[1])k^{3}+2{\left(a_{s}-2\right)}^{2}(\frac{3}{2}a_{s}-a_{s}D[1])k^{2}\\ +{\left(a_{s}-2\right)}^{2}(\frac{3}{2}a_{s}+\frac{3}{2}a_{s}-a_{s}D[1]-a_{s}D[2])k^{2}+(a_{s}-2)(\frac{3}{2}a_{s}-a_{s}D[1])k\\ +(a_{s}-2)(\frac{3}{2}a_{s}+\frac{3}{2}a_{s}-a_{s}D[1]-a_{s}D[2])k+(a_{s}-2)(\frac{3}{2}a_{s}+\frac{3}{2}a_{s}+\frac{3}{2}a_{s}-a_{s}D[1]-a_{s}D[2]-a_{s}D[3])k\\ +4\cdot \frac{3}{2}a_{s}-a_{s}(D[1]+D[2]+D[3]+D[4])\} \end{array}.$$

After i>5, the first-order DLL could be expressed as follows:

(A6) $$\tau [i]=\tau [i-1]+k\{-(2a_{s}+2)\tau [i-1]+2a_{s}d[i-1]\}.$$

If τ is developed until i=n, SPE is complete. SPE has the following generalized form like:

(A7) $$\tau [n]=\sum_{i=1}^{n}g_{i,n}(a_{s})k^{i},$$

(A8) $$g_{n,n}(a_{s})={\left(-1\right)}^{m}{}_{m}C_{m}{\left(2a_{s}+2\right)}^{m}{\left(a_{s}-2\right)}^{l-1}(\frac{3}{2}a_{s}-ad[1]),$$

(A9) $$\begin{array}{l} g_{n-1,n}(a_{s})={\left(-1\right)}^{m-1}{}_{m}C_{m-1}{\left(2a_{s}+2\right)}^{m-1}{\left(a_{s}-2\right)}^{l-1}(\frac{3}{2}a_{s}-a_{s}d[1])\;\\ +{\left(-1\right)}^{m}3_{m}C_{m}{\left(2a_{s}+2\right)}^{m}{\left(a_{s}-2\right)}^{l-2}(\frac{3}{2}a_{s}-a_{s}d[1])\\ +{\left(-1\right)}^{m}3_{m}C_{m}{\left(2a_{s}+2\right)}^{m}{\left(a_{s}-2\right)}^{l-2}\{2\frac{3}{2}a-a(d[1]+d[2])\} \end{array},$$

(A10) $$\begin{array}{l} g_{n-2,n}(a_{s})={\left(-1\right)}^{m-2}{}_{m}C_{m-2}{\left(2a_{s}+2\right)}^{m-2}{\left(a-2\right)}^{l-1}(\frac{3}{2}a_{s}-a_{s}d[1])\\ \;+{\left(-1\right)}^{m-1}3_{m}C_{m-1}{\left(2a_{s}+2\right)}^{m-1}{\left(a_{s}-2\right)}^{m-2}(\frac{3}{2}a_{s}-a_{s}d[1])\\ \;+{\left(-1\right)}^{m-1}2_{m}C_{m-1}{\left(2a_{s}+2\right)}^{m-1}{\left(a_{s}-2\right)}^{l-2}\{2\frac{3}{2}a_{s}-a_{s}(d[1]+d[2])\}\\ \;+{\left(-1\right)}^{m}3_{m}C_{m}{\left(2a_{s}+2\right)}^{m}{\left(a_{s}-2\right)}^{l-3}(\frac{3}{2}a_{s}-a_{s}d[1])+{\left(-1\right)}^{m}2_{m}C_{m}{\left(2a_{s}+2\right)}^{m}{\left(a_{s}-2\right)}^{l-3}\{2\frac{3}{2}a_{s}-a_{s}(d[1]+d[2])\}\\ \;+{\left(-1\right)}^{m}{}_{m}C_{m}{\left(2a_{s}+2\right)}^{m}{\left(a_{s}-2\right)}^{l-3}\{3\frac{3}{2}a-a(d[1]+d[2]+d[3])\} \end{array}.$$

## Appendix B

To derive the SPE, each DLL order could be expressed as follows:

(A11) $$\begin{array}{l} \tau [n+1]=\tau [n]-\omega_{0}\cdot T\cdot \Delta \tau [n]\\ \;=\tau [n]-K_{1}\cdot \Delta \tau [n]\\ \; \end{array}.$$

(A12) $$\begin{array}{l} \tau [n+1]=\tau [n]-\{{\left(\omega_{1}\cdot T\right)}^{2}\cdot \Delta \tau [n]+a_{2}\cdot \omega_{1}\cdot T\cdot \Delta \tau [n]\}+\tau_{1}[n]\cdot T\\ \;=\tau [n]-(k_{2}\cdot \Delta \tau [n]+k_{3}\cdot \Delta \tau [n])+\tau_{1}[n]\cdot T\\ \;=\tau [n]-(k_{2}+k_{3})\Delta \tau [n]+\tau_{1}[n]\cdot T\\ \;=\tau [n]-K_{2}\cdot \Delta \tau [n]+\tau_{1}[n]\cdot T\\ \; \end{array}$$

(A13) $$\begin{array}{l} \tau [n+1]=\tau [n]-\{{\left(\omega_{2}\cdot T\right)}^{3}\cdot \Delta \tau [n]+{\left(a_{3}\cdot \omega_{2}\cdot T\right)}^{2}\Delta \tau [n]\\ \;+(b_{3}\cdot \omega_{2}\cdot T)\cdot \Delta \tau [n]\}+\tau_{2}[n]\cdot T^{2}+\tau_{3}[n]\cdot T\\ \;=\tau [n]-(k_{4}\Delta \tau [n]+k_{5}\Delta \tau [n]+k_{6}\Delta \tau [n])+\tau_{2}[n]\cdot T^{2}+\tau_{3}[n]\cdot T\\ \;=\tau [n]-(k_{4}+k_{5}+k_{6})\Delta \tau [n]+\tau_{2}[n]\cdot T^{2}+\tau_{3}[n]\cdot T\\ \;=\tau [n]-K_{3}\cdot \Delta \tau [n]+\tau_{2}[n]\cdot T^{2}+\tau_{3}[n]\cdot T\\ \; \end{array}$$

Equations (A11) to (A13) are the DLL in case of 1st, 2nd, and 3rd order, respectively. $\omega_{0}$ is the 1st order filter value. $\omega_{1}$ and $a_{2}$ are the 2nd order filter values. $\omega_{2}$, $a_{3}$ and $b_{3}$ are the 3rd order filter values. $K_{1}$, $K_{2}$ and $K_{3}$ are the SPE coefficients with respect to the DLL order, respectively. SPE coefficients are determined through the filter setting. Also, $\tau_{1}[n]$ in Equation (A12) can be derived using Equation (A11). And $\tau_{2}[n]$ and $\tau_{3}[n]$ in Equation (A13) can be derived using Equation (A11) and Equation (A12), respectively. This derivation shows that the SPE could be derived from any DLL order.

## References

[1] Psiaki M.L., Humphreys T.E. (2016). GNSS Spoofing and Detection. Proc. IEEE.

[2] Cavaleri A., Motella B., Pini M., Fantino M. Detection of spoofed GPS signals at code and carrier tracking level. Proceedings of the 2010 5th ESA Workshop on Satellite Navigation Technologies and European Workshop on GNSS Signals and Signal Processing (NAVITEC).

[3] Tippenhauer N.O., Pöpper C., Rasmussen K.B., Capkun S. On the requirements for successful GPS spoofing attacks. Proceedings of the 18th ACM Conference on Computer and Communications Security.

[4] Hui H., Na W. A study of GPS jamming and anti-jamming. Proceedings of the 2009 2nd International Conference on Power Electronics and Intelligent Transportation System (PEITS).

[5] Ng Y., Gao G.X. Mitigating jamming and meaconing attacks using direct GPS positioning. Proceedings of the 2016 IEEE/ION Position, Location and Navigation Symposium (PLANS).

[6] Kerns A.J., Shepard D.P., Bhatti J.A., Humphreys T.E. (2014). Unmanned aircraft capture and control via GPS spoofing. J. Field Robot..

[7] Jafarnia-Jahromi A., Broumandan A., Nielsen J., Lachapelle G. (2012). GPS vulnerability to spoofing threats and a review of antispoofing techniques. Int. J. Navig. Obs..

[8] Humphreys T.E., Ledvina B.M., Tech V., Psiaki M.L., Hanlon B.W.O., Kintner P.M. Assessing the Spoofing Threat: Development of a Portable GPS Civilian Spoofer. Proceedings of the 21st International Technical Meeting of the Satellite Division of The Institute of Navigation (ION GNSS 2008).

[9] Shepard D.P., Bhatti J.A., Humphreys T.E. (2012). Drone Hack: Spoofing Attack Demonstration on a Civilian Unmanned Aerial Vehicle. GPS World.

[10] Bhatti J., Humphreys T.E. (2017). Hostile Control of Ships via False GPS Signals: Demonstration and Detection. Navig. J. Inst. Navig..

[11] Wang K., Chen S., Pan A. (2015). Time and Position Spoofing with Open Source Projects.

[12] Wang F., Li H., Lu M. (2017). GNSS spoofing detection and mitigation based on maximum likelihood estimation. Sensors.

[13] Psiaki M.L., O’Hanlon B.W., Bhatti J.A., Shepard D.P., Humphreys T.E. (2013). GPS spoofing detection via dual-receiver correlation of military signals. IEEE Trans. Aerosp. Electron. Syst..

[14] Manfredini E.G., Dovis F. (2016). On the use of a feedback tracking architecture for satellite navigation spoofing detection. Sensors.

[15] Liu K., Wu W., Wu Z., He L., Tang K. (2018). Spoofing detection algorithm based on pseudorange differences. Sensors.

[16] Shafiee E., Mosavi M.R., Moazedi M. (2018). Detection of Spoofing Attack using Machine Learning based on Multi-Layer Neural Network in Single-Frequency GPS Receivers. J. Navig..

[17] Daneshmand S., Jafarnia-jahromi A., Broumandan A., Lachapelle G. A Low-Complexity GPS Anti-Spoofing Method Using a Multi-Antenna Array. Proceedings of the 25th International Technical Meeting of The Satellite Division of the Institute of Navigation.

[18] Broumandan A., Lachapelle G. (2018). Spoofing Detection Using GNSS/INS/Odometer Coupling for Vehicular Navigation. Sensors.

[19] Perdue L., Sasaki H., Fischer J. Testing GNSS Receivers to Harden Against Spoofing Attacks. Proceedings of the International Symposium on GNSS 2015.

[20] Ma C., Lachapelle G., Cannon M.E. (2003). Implementation of a Software GPS Receiver. Architecture.

